# IBS and SIBO: Gut Microbiota, Pathophysiology, and Non-Pharmacological Interventions

**DOI:** 10.3390/antibiotics15030251

**Published:** 2026-02-27

**Authors:** Jelena Šuran, Nikola Pavlović, Joško Božić, Marko Kumrić, Katarina Vukojević, Natalija Filipović, Božo Radić

**Affiliations:** 1 Apiotix Technologies d.o.o., Hrvojeva 12, 21000 Split, Croatia; 2 Department of Pathophysiology, University of Split School of Medicine, 21000 Split, Croatia; josko.bozic@mefst.hr (J.B.);; 3 Laboratory for Cardiometabolic Research, University of Split School of Medicine, 21000 Split, Croatia; 4 Department of Cardiovascular Diseases, University Hospital of Split, 21000 Split, Croatia; 5 Department of Anatomy, Histology and Embryology, University of Split School of Medicine, 21000 Split, Croatia; katarina.vukojevic@mefst.hr (K.V.); natalija.filipovic@mefst.hr (N.F.); 6 Mediterranean Institute for Life Sciences, University of Split, 21000 Split, Croatia

**Keywords:** irritable bowel syndrome, SIBO, gut–brain axis, breath test, probiotics, motility

## Abstract

Irritable bowel syndrome (IBS) and small intestinal bacterial overgrowth (SIBO) share symptoms such as abdominal pain, bloating, and altered bowel habits. Both are linked to dysbiosis and gut–brain axis dysfunction. IBS is a multifactorial disorder characterized by abnormal motility, visceral hypersensitivity, low-grade inflammation, and alterations in the microbiota. In contrast, SIBO is defined by excessive bacterial colonization of the small intestine that can mimic or worsen IBS symptoms. Gut microbes and their metabolites influence motility, immune activation, barrier integrity, and gas production; methanogen overgrowth is associated with constipation-predominant presentations, while hydrogen- and hydrogen sulfide-related pathways may contribute to diarrhea and bloating. Because recurrent or empiric antibiotic use is common—particularly in suspected SIBO—yet carries risks of resistance, microbiome disruption, and relapse, there is a strong rationale to prioritize effective non-antibiotic strategies. Accordingly, this review synthesizes current evidence on IBS/SIBO pathophysiology and microbiota interactions. It evaluates non-pharmacological interventions including dietary approaches, probiotics/prebiotics, herbal therapies, and mind–body treatments (e.g., cognitive behavioral therapy and gut-directed hypnotherapy). We emphasize an integrative framework that supports symptom control and quality of life while helping reduce unnecessary antibiotic exposure.

## 1. Introduction

Irritable bowel syndrome (IBS) is a chronic disorder of gut–brain interaction characterized by recurrent abdominal pain associated with altered bowel habits, in the absence of identifiable structural abnormalities [1]. It affects an estimated 5–10% of the global population and is more common in women, imposing a significant burden on quality of life and healthcare resources [2]. Small intestinal bacterial overgrowth (SIBO), defined as an excessive microbial population in the small intestine (traditionally >103 CFU/mL in aspirate culture), can produce GI symptoms overlapping with IBS, such as bloating, abdominal discomfort, diarrhea, and/or constipation [3]. A positive carbohydrate breath test (hydrogen and/or methane) is a practical diagnostic proxy for SIBO, although diagnostic standards vary [4,5]. Notably, a subset of IBS patients exhibits positive breath tests suggestive of SIBO, leading to the hypothesis of an etiological overlap. Early studies reported SIBO in up to 60–78% of IBS cases based on lactulose breath testing [5,6,7], though later investigations using more rigorous methods found lower prevalences (e.g., 4–20%) [6,7]. While the precise causal relationship between SIBO and IBS remains debated, their frequent clinical co-occurrence and overlapping symptom profiles warrant an integrated therapeutic approach.

Given the complex and multifactorial nature of IBS, management is typically multi-pronged. Conventional pharmacological treatments (e.g., laxatives, antispasmodics, visceral analgesics, antibiotics) often provide incomplete relief or carry side effects, prompting many patients to seek non-pharmacological options [8]. Diet and lifestyle modifications, as well as other non-drug therapies, are now considered integral to standard care for IBS, alongside medications [8]. Moreover, increasing evidence implicating the gut microbiota in IBS/SIBO has spurred interest in interventions that modulate microbial communities.

Here we provide a comprehensive overview of IBS and SIBO, focusing on their pathophysiology and the role of the gut microbiota. We review evidence-based nonpharmacological interventions, including dietary strategies, probiotics, herbal therapies, and mind–body approaches, that target these conditions. Despite the growing literature on IBS and SIBO, guidance remains fragmented—microbial mechanisms, motility, and dietary triggers are often discussed in isolation. We bring these factors together and treat IBS and SIBO as a connected spectrum for a subset of patients, where symptoms can be addressed through managing dysbiosis, barrier and immune signaling, and gut–brain interactions. We acknowledge that evidence for a direct causal link between SIBO and IBS is limited and heterogeneous. However, their interface is clinically meaningful because: (1) they share overlapping symptoms and pathophysiological mechanisms (dysbiosis, motility dysfunction, visceral hypersensitivity, immune activation); (2) interventions targeting the small intestinal microbiota demonstrate efficacy in IBS even when strict SIBO criteria are not met; and (3) in clinical practice, distinguishing between these conditions is often challenging, making an integrated management framework valuable regardless of which condition is primary.

## 2. Pathophysiology of IBS and SIBO

### 2.1. IBS Pathophysiology

IBS is a multifactorial syndrome characterized by the dysregulation of the brain–gut axis, abnormal gastrointestinal (GI) motility, visceral hypersensitivity, immune activation, and alterations in intestinal microbiota [9,10]. Patients often exhibit heightened pain perception to gut distension (visceral hyperalgesia) and dysfunction in the communication between the central nervous system and the enteric nervous system (i.e., brain-gut axis dysregulation) [10]. Aberrant motility patterns are common, for instance, accelerated transit in diarrhea-predominant IBS (IBS-D) and delayed transit in constipation-predominant IBS (IBS-C), contributing to changes in bowel habits. Low-grade inflammation and immune activation have also been noted in some IBS patients (e.g., increased mucosal mast cells and T lymphocytes), which may disrupt the epithelial barrier and neural signaling in the gut [11,12]. A significant subset of IBS cases arises after an acute gastroenteritis, termed post-infectious IBS (PI-IBS). In PI-IBS, an initial infection triggers persistent alterations, including increased intestinal permeability, changes in enteroendocrine cells, and an elevated immune response, which can lead to chronic IBS symptoms [11,13]. Long-term changes in the gut ecosystem following infection are also thought to play a role [13,14].

### 2.2. SIBO Pathophysiology

SIBO is characterized by an abnormally high bacterial load in the small intestine, often involving bacteria more typical of the colon [3]. Several protective mechanisms typically limit small bowel bacterial counts, including gastric acid, bile, pancreatic enzymes, an intact ileocecal valve, and especially gastrointestinal motility (migrating motor complex activity during fasting), which sweeps bacteria distally [3]. When these defenses are compromised, bacteria can accumulate proximally. Clinical interventional evidence also supports a symptom link between SIBO and IBS, as eradication of SIBO has been associated with improvement of IBS symptoms [15]. For example, diminished motility or impaired migrating motor complexes are strongly associated with SIBO development [5]. Conditions such as diabetes, scleroderma, intestinal strictures, or surgeries, as well as chronic proton pump inhibitor (PPI) use (which reduces gastric acidity), are recognized risk factors for SIBO [4]. In addition, *Helicobacter pylori* infection represents another potential upstream modifier of host defenses, as chronic infection may alter acid secretion and thereby compromise one of the primary barriers limiting proximal bacterial load [16]. In line with this concept, Wang et al. reported an increased prevalence of SIBO and IMO among *H. pylori*–infected individuals, with improved breath-test outcomes and symptom scores following eradication therapy [16].

SIBO can itself further damage the brush border and impair nutrient absorption, perpetuating a vicious cycle of malabsorption, mucosal inflammation, and motility changes. Excessive fermentation by small bowel bacteria produces gases (hydrogen, methane, and hydrogen sulfide) that can provoke bloating, pain, and altered stool consistency. The type of gas produced has clinical relevance: hydrogen overproduction is often linked to diarrhea (H_2_-SIBO), whereas methane production (by *Methanobrevibacter archaea*) is associated with constipation. Recent reviews emphasize that methane is produced by methanogenic archaea, such as Methanobrevibacter smithii, rather than by bacteria, and is clinically relevant to motility phenotypes [17]. Methanogenic archaea consume hydrogen to produce methane, and methane has a direct effect of slowing intestinal transit [18]. Indeed, methane-positive breath tests correlate with constipation severity in IBS [5], and the concept of intestinal methanogen overgrowth (IMO) has been introduced to distinguish methane-predominant cases, since these organisms can colonize both the small and large intestine [3,18]. Hydrogen sulfide (H_2_S), a third gas detectable by newer breath tests, has been linked to diarrhea-predominant symptoms, but H_2_S-predominant patterns appear relatively uncommon and diagnostic thresholds remain less standardized; therefore, the term intestinal sulfide overproduction (ISO) has been proposed to describe this phenotype [3,4,17].

Importantly, SIBO differs from IBS in that it is more directly linked to failure of host mechanisms that normally restrain microbial density in the proximal small intestine. Whereas IBS is defined clinically within the spectrum of disorders of gut–brain interaction [1], SIBO more often occurs in the setting of identifiable predisposing factors—particularly impaired migrating motor complex activity, anatomical abnormalities that promote stasis (e.g., strictures, blind loops, post-surgical changes), systemic diseases affecting motility (e.g., diabetes, scleroderma), or medication exposures that alter luminal conditions (e.g., chronic PPI use) [3,17]. SIBO can also be accompanied by maldigestion and malabsorption-related features, including bile acid deconjugation, carbohydrate malabsorption, and, in some cases, nutrient deficiencies, which are not defining characteristics of IBS [3]. Finally, diagnostic standards for SIBO remain heterogeneous: breath tests are widely used but represent indirect proxies influenced by transit and fermentation patterns, complicating direct comparisons across cohorts [4].

### 2.3. Intersections Between IBS and SIBO

There is considerable symptomatic overlap between IBS and SIBO, and growing evidence suggests they are interconnected in some patients. Importantly, inflammatory bowel diseases (IBDs), such as ulcerative colitis and Crohn’s disease, can present with overlapping symptoms, including abdominal pain, bloating, diarrhea, and altered bowel habits [1,3]. This overlap is clinically relevant because SIBO and IMO can co-occur in IBD, particularly Crohn’s disease with small-bowel involvement, where inflammation, strictures, prior ileocecal resection, or altered motility promote stasis and bacterial/archaeal overgrowth [1,3]. Therefore, persistent bloating, gas, or diarrhea in IBD, especially during remission, should be interpreted in the context of inflammatory activity, structural risk factors, and possible coexisting SIBO [1,3].

It has been hypothesized that SIBO may act as a trigger or contributing factor for IBS symptoms in a subset of individuals, though the directionality and causality of this relationship remain areas of active investigation. Bacterial overgrowth in SIBO can lead to carbohydrate malfermentation and gas production, which could contribute to bloating and pain that mimic or potentially initiate IBS symptoms. Bacterial metabolites and endotoxins in SIBO can also induce low-grade inflammation and visceral hypersensitivity, aligning with IBS pathophysiology. Some researchers hypothesize that IBS with bloating may often be SIBO-related [19]. Clinical studies support an association: a meta-analysis of case–control studies found that SIBO was significantly more prevalent in IBS patients than in controls, although estimates varied widely depending on the diagnostic method [6]. As noted, non-invasive breath tests in IBS show a high positivity rate in some cohorts. This is particularly evident in United States-based cohorts, where the frequent use of the lactulose breath test (LBT) results in a SIBO prevalence of 62.3%, and in diarrhea-predominant (IBS-D) populations, where positivity rates reach 35.5%, whereas constipation-predominant (IBS-C) cohorts are specifically distinguished by a nearly three-fold higher prevalence of methane-positive tests (25.3%) compared to the diarrhea subtype. In contrast, direct small intestine aspirate cultures (the gold standard) reveal a more modest overgrowth rate, highlighting that diagnostic criteria influence prevalence figures [3,7]. These systematic reviews also highlight significant methodological heterogeneity across studies and conclude that the overall quality of evidence linking SIBO causally to IBS remains limited [6,7]. The relationship appears more nuanced than simple causation, likely involving bidirectional influences and shared pathophysiological mechanisms.

Nonetheless, improvements in IBS symptoms with antibiotic therapy in some trials provide indirect evidence of a role for the microbiota. A landmark placebo-controlled study demonstrated that the non-absorbable antibiotic rifaximin significantly improved symptoms in IBS patients without constipation. However, this specific trial did not confirm the presence or eradication of SIBO in its participants [20]. This resulted in the clinical approval of rifaximin for the treatment of IBS-D. However, evidence specifically linking SIBO eradication to symptom relief is found in other studies, which show that normalizing breath tests via antibiotic therapy correlates with significant clinical improvement in IBS symptoms [15]. Thus, SIBO and IBS likely share a bidirectional relationship where microbial perturbations and motility changes contribute to the genesis of gastrointestinal symptoms [17]. The physiological alterations inherent in IBS may create a permissive environment for SIBO development [17]. In constipation-predominant IBS, the presence of methanogenic archaea produces methane gas, which has been shown to slow intestinal transit and augment small intestinal contractile activity, potentially fostering an environment conducive to further bacterial overgrowth [18]. While these mechanisms provide a plausible link between IBS and SIBO, longitudinal data confirming this causal direction remain limited, and the relationship is likely defined by complex, bidirectional feedback loops.

IBS-oriented therapies may help SIBO patients due to symptoms overlapping with abdominal pain, bloating, and altered bowel movements. Although these symptoms start from different causes, they are driven by convergent mechanisms, such as luminal distension leading to bloating and pain, visceral hypersensitivity (pain even with normal distension) [9,10], motility disruption (leads to diarrhea or constipation and stasis) [3,17,19], and altered immune signaling followed by low-grade inflammation and permeability changes [11,12]. Thus, interventions that reduce fermentation or inhibit gut–brain amplification through different approaches can improve symptoms regardless of the initiating factor.

## 3. The Gut Microbiota in IBS and SIBO

The composition and activity of the gut microbiota have emerged as key factors in IBS pathogenesis and a defining element of SIBO. Under normal conditions, the small intestine harbors a relatively low bacterial biomass, distinct from the dense, diverse microbial community of the colon. In IBS, accumulating evidence indicates a state of dysbiosis—an imbalance in the gut microbial community, compared to healthy individuals [21]. Studies using stool DNA sequencing have found that IBS patients often have reduced bacterial diversity and altered community profiles [22]. For instance, one study reported that patients with diarrhea-predominant IBS had lower overall microbial biodiversity, with an overrepresentation of specific genera, such as *Bacteroides* and *Prevotella*, and a relative depletion of beneficial anaerobes [22]. A common finding is a decrease in populations of *Lactobacillus* and *Bifidobacterium* (often considered beneficial commensals) alongside an increase in facultative or potentially pathogenic taxa, such as certain Proteobacteria or Firmicutes that produce gas and irritants—a pattern consistent with the dysbiosis observed in IBS [23,24]. Some IBS patients show an overabundance of methanogenic archaea or sulfate-reducing bacteria, which could influence gas production and symptoms [25].

Additionally, imbalances in microbial metabolites are central to the pathogenesis of IBS and SIBO. Microbiota-derived metabolites—including short-chain fatty acids (SCFAs), choline, bile acids (BAs), polyamines, and indoles—are increasingly recognized as key mediators of gut–immune and gut–brain signaling in IBS [23]. Beyond the well-documented reduction in butyrate-producing bacteria and anti-inflammatory short-chain fatty acids (SCFAs) [22], alterations in bile acid metabolism and tryptophan signaling have emerged as important contributors to visceral hypersensitivity and immune activation [21,23]. In IBS-D, enrichment of primary bile acids—resulting from impaired microbial deconjugation—may stimulate secretion and motility via bile acid receptor signaling pathways [21]. Dysbiotic patterns may shift tryptophan metabolism away from indole production (supporting epithelial integrity) toward the kynurenine pathway, promoting low-grade mucosal inflammation [23]. In SIBO, higher proportions of hydrogenotrophic microbes, including methanogens and sulfate-reducing bacteria, further alter gas profiles by producing methane or hydrogen sulfide (H_2_S), both of which are implicated in gastrointestinal dysmotility and symptom generation [17,21]. Importantly, these metabolic signatures are not uniform; IBS is highly heterogeneous, and distinct microbiome signatures are increasingly linked to specific subtypes and symptom severity [22,24].

SIBO, by definition, is a form of dysbiosis characterized by microbial overgrowth in the small intestine. While SIBO and IBS frequently co-occur, it remains unclear whether SIBO is a cause, consequence, or parallel phenomenon in most IBS cases. What is evident, however, is that both conditions involve dysbiosis and share overlapping mechanisms of symptom generation. The microbiota of SIBO is less well characterized than that of the colon due to sampling difficulties. Still, culture and sequencing data indicate that common SIBO organisms include aerobes and facultative anaerobes that generally reside in the colon or oral cavity, such as *Escherichia coli*, *Klebsiella*, *Enterococcus*, and *Streptococcus* [17]. When present in excess in the small bowel, these organisms can displace the normal sparse flora and disrupt nutrient processing. They ferment carbohydrates to produce hydrogen gas (leading to H_2_-Type SIBO) or, in the presence of methanogens, hydrogen is converted into methane (CH_4_) [3]. The recognition that methanogenic archaea drive methane production has prompted reclassification of methane-positive cases as intestinal methanogen overgrowth (IMO), since methanogens may colonize both the small and large intestine and methane positivity is not specific to small-bowel overgrowth [3,4,17]. Methane producers have been explicitly linked to constipation-predominant IBS, supporting a mechanistic overlap in which methanogen-associated methane production contributes to motility changes that manifest as IBS-C [5,18]. Meanwhile, emerging research on hydrogen sulfide (H_2_S)-producing microbes suggests a possible link to diarrhea symptoms [17]. However, routine clinical testing for H_2_S-SIBO is not yet standard [3,4].

Alterations in the microbiota can contribute to IBS symptoms through multiple mechanisms. First, gut bacteria interact with the host’s immune system: an overgrowth or imbalance can activate mucosal immune responses, mast cells, and cytokine release, contributing to the low-grade inflammation observed in some IBS [23]. Second, bacteria and their metabolites can influence intestinal barrier function—for example, dysbiosis may increase epithelial permeability (“leaky gut”), leading to greater luminal antigen penetration and neural sensitization. Some patients with IBS, particularly IBS-D, show evidence of increased colonic epithelial permeability and mucosal dysfunction, potentially mediated by microbiota-derived factors [26]. Third, bacterial metabolites such as SCFAs, bile acid derivatives, tryptophan metabolites, and gases can affect gut sensory and motor functions. SCFAs (like butyrate, propionate) normally nourish colonocytes and have anti-inflammatory effects, so a reduction in butyrate-producers in IBS could adversely affect gut function [25,27]. Excessive production of gas and osmotic metabolites from malfermentation can cause luminal distension (bloating) and abnormal motility (diarrhea or constipation). Furthermore, bacteria can produce toxins or neurotransmitter-like chemicals (e.g., tryptophan metabolites, including indole derivatives, or histamine) that influence enteric nervous system signaling, potentially contributing to changes in pain or motility. The involvement of the gut–brain axis is also evident, where microbial dysbiosis may alter signals along the vagus nerve or modulate tryptophan metabolism and serotonin availability, thereby impacting mood and pain perception [28]. This is in line with the observation that a subset of IBS patients has comorbid psychological distress. Also, preclinical studies have shown that transplanting dysbiotic microbiota can induce anxiety and visceral hypersensitivity in animal models [29]. This suggests the microbiota may be a mediator between stress and gut dysfunction, though human data are still evolving.

In summary, IBS can be viewed, in part, as a microbiome-associated disorder, and SIBO represents one extreme of gut microbiota alteration, localized to the small intestine. Both conditions underscore the significance of microbial ecology in gastrointestinal health. This understanding has paved the way for therapies aimed at correcting dysbiosis. Antibiotics, such as rifaximin, can reduce bacterial overgrowth and have demonstrated efficacy in IBS-D [30], suggesting that modulating the small intestinal microbiota can provide symptomatic benefit even when the causal relationship between bacterial overgrowth and IBS symptoms remains incompletely understood. More physiologically, dietary modifications alter nutrient flow to the microbiota. Probiotics and prebiotics aim to restore microbial balance, and fecal microbiota transplantation (FMT) has been explored as a means to replace dysbiotic communities with a healthy microbiome. Notably, trials of FMT in IBS have yielded mixed results—some showing significant symptom relief and microbiota shifts [31], others showing no benefit over placebo [32], underscoring that simply altering the microbiota does not guarantee symptom resolution, and that donor selection, dosing, and IBS subtype may determine outcomes. Nevertheless, the gut microbiome remains a promising therapeutic target in IBS and SIBO. In the following sections, we discuss established and emerging non-pharmacological interventions that leverage diet, microbiota modulation, and other modalities to manage IBS and SIBO (Table 1).

## 4. Therapeutic Interventions: Non-Pharmacological Approaches

**Figure 1:**
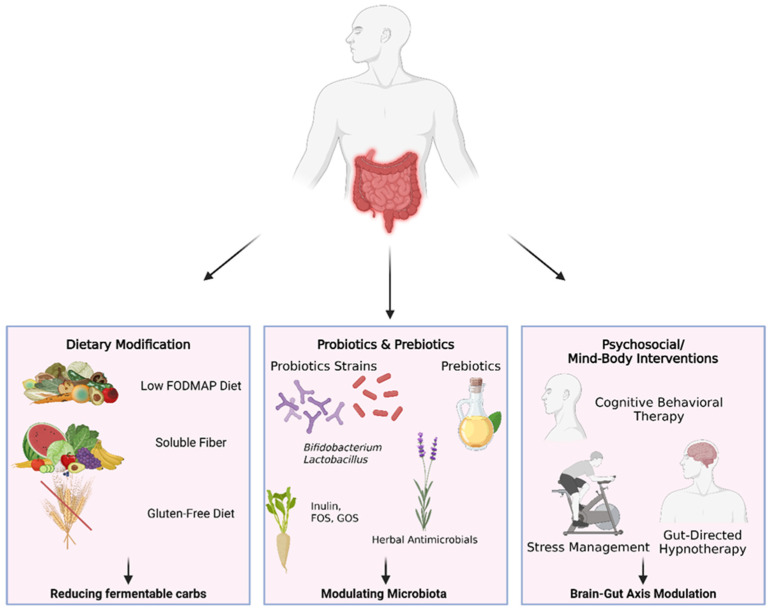
Non-pharmacological interventions targeting pathophysiological mechanisms in IBS and SIBO. This schematic illustrates key non-pharmacological strategies for addressing gut-related symptoms in irritable bowel syndrome (IBS) and small intestinal bacterial overgrowth (SIBO). The central image highlights the gastrointestinal tract as the primary target, with three major intervention domains shown below. Dietary modification focuses on reducing fermentable substrates through approaches such as a low-FODMAP diet, soluble fiber supplementation, and gluten-free diets, thereby decreasing excessive fermentation and symptom generation. Probiotics and prebiotics aim to modulate the intestinal microbiota by using specific probiotic strains (e.g., *Bifidobacterium* and *Lactobacillus*), prebiotic fibers (inulin, fructooligosaccharides [FOS], galactooligosaccharides [GOS]), and selected herbal antimicrobials. Psychosocial and mind–body interventions target dysregulation of the brain–gut axis and include cognitive behavioral therapy, stress management techniques, and gut-directed hypnotherapy. Collectively, these interventions act on complementary mechanisms—dietary fermentation, microbial composition, and brain–gut interactions—to alleviate symptoms in IBS and SIBO. Created in BioRender. Vukojevic, K. (2026) https://BioRender.com/76t92qr (accessed on 3 November 2025).

Effective management of IBS (and SIBO-related gastrointestinal symptoms) often requires a holistic approach that goes beyond conventional medications. Non-pharmacological interventions are now recognized as first-line or adjunct therapies that can address the various pathophysiological dimensions of IBS, including dysregulated motility, visceral sensitivity, microbiota imbalance, and psychosocial factors, with typically fewer side effects. For SIBO, non-antibiotic strategies can be important for patients who cannot tolerate antibiotics or have recurrent overgrowth. Below, we review key categories of non-pharmacological interventions, including dietary modifications, probiotics and prebiotics, herbal and nutraceutical therapies, and psychosocial or mind–body interventions. These approaches can be tailored and combined based on individual patient triggers and predominant symptom profiles (Figure 1).

### 4.1. Dietary Interventions

One of the most evidence-backed dietary strategies for IBS is the low FODMAP (fermentable oligosaccharides, disaccharides, monosaccharides, and polyols) diet. FODMAPs are a group of short-chain carbohydrates (e.g., fructans, galacto-oligosaccharides, lactose, excess fructose, sorbitol, mannitol) that are poorly absorbed in the small intestine and thus can be fermented by colonic bacteria, producing gas and drawing water into the lumen [33]. In many IBS patients, especially those with bloating and diarrhea, reducing dietary FODMAP intake leads to improvement in symptoms. Randomized controlled trials have demonstrated that a low FODMAP diet significantly reduces IBS global symptoms, abdominal pain, and bloating compared to standard diets [34,35]. A meta-analysis reported an overall response rate around 70% with the low FODMAP diet in IBS, though response definitions vary [36]. The mechanism of benefit is believed to be reduced colonic fermentation, reduced gas production, and reduced luminal distension. In SIBO, however, the overgrowth of bacteria in the small intestine can ferment FODMAPs prematurely (before they reach the colon), producing gas and symptoms in the upper GI tract. By restricting FODMAPs, the diet reduces the substrate available to both small intestinal overgrowth bacteria and colonic microbiota, thereby alleviating bloating and discomfort in both compartments [37]. The low FODMAP diet was well tolerated and reduced reported gastrointestinal symptoms even in patients with SIBO [37]. However, studies on the efficacy of low FODMAP in SIBO are scarce.

The low FODMAP diet is implemented in phases: an initial strict restriction of high-FODMAP foods for typically 2–6 weeks, followed by a structured reintroduction phase to identify specific triggers, and finally, personalization to the most tolerable diet in the long term [38]. Foods rich in FODMAPs that are curtailed include certain fruits (e.g., apples, pears, mango), high-lactose dairy, wheat and rye, legumes, onions and garlic, and artificial sweeteners like sorbitol, among others [39]. While effective for many, the low FODMAP diet comes with nutritional and practical considerations. It can be restrictive and challenging to follow without guidance; dietitian supervision is recommended to ensure nutritional adequacy and to liberalize the diet as much as possible after the elimination phase. Another concern is that strict long-term FODMAP restriction may adversely affect the gut microbiota—FODMAPs are naturally occurring prebiotics (fibers) that feed beneficial bacteria like *Bifidobacterium* [40]. Studies have shown that while on a low FODMAP diet, *Bifidobacterium* levels tend to decrease (since many fibers that stimulate bifidobacteria are cut out) [39]. For this reason, experts emphasize the importance of reintroducing as many FODMAPs as tolerated and not staying on the complete elimination indefinitely [40]. Overall, when used appropriately, the low FODMAP diet is a valuable tool for controlling IBS symptoms and is included in several clinical guidelines as a recommended therapy [1]. In SIBO, it serves primarily as symptomatic management to reduce fermentation byproducts; it may be combined with antibiotic or probiotic therapy in an integrative approach.

Dietary fiber content can significantly influence IBS symptoms, and adjustments in fiber intake are a simple yet effective way to manage these symptoms. Insoluble, coarse fiber (such as wheat bran) has historically been recommended for constipation; however, in IBS, it can sometimes exacerbate gas and discomfort. In contrast, soluble fiber (psyllium husk/ispaghula, oats, pectin) tends to be better tolerated and beneficial. A systematic review and meta-analysis found that soluble fiber (psyllium) significantly improved global IBS symptoms. In contrast, insoluble fiber had no benefit and may even have worsened symptoms [41]. Psyllium supplements improve stool consistency in constipation and can also bind water, reducing urgency in diarrhea, all while avoiding excessive fermentation in the colon. Accordingly, clinical guidelines recommend supplementation with approximately 20–30 g of psyllium or similar soluble fiber per day as a first-line measure in IBS [42]. Fiber is also important for overall gut health and feeds commensal bacteria. Interestingly, even on a low-FODMAP diet, ensuring adequate soluble fiber (from low-FODMAP sources like oats, kiwi, or psyllium) can help mitigate adverse microbiota impacts by providing a fermentable substrate for beneficial microbes. In SIBO management, fiber plays a nuanced role. While fiber can promote healthy microbiota in the long run, introducing fiber when SIBO is active may be contraproductive, as bacteria in the small intestine can ferment fiber prematurely, leading to early gas production and bloating [38]. Some SIBO protocols advise delaying high-dose fiber until after initial antimicrobial therapy. Nonetheless, partially hydrolyzed guar gum (a soluble fiber) has been used alongside antibiotics in SIBO to improve outcomes, and one trial suggested adding guar gum to rifaximin increased SIBO eradication rates, possibly by enhancing small bowel motility and flushing effect [43]. Thus, fiber should be individualized—many IBS/SIBO patients benefit from a gradual increase in soluble fiber to regulate bowels, with careful attention to tolerance.

**Figure 2:**
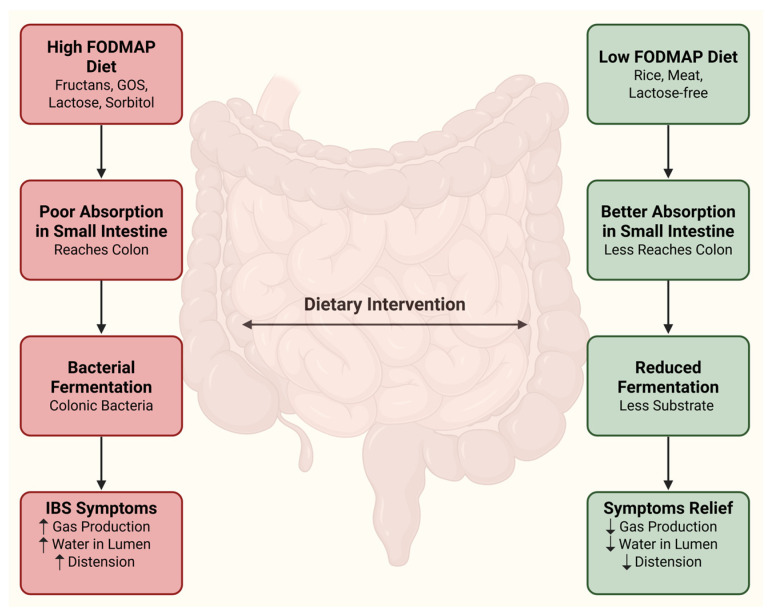
The figure depicts the proposed mechanism by which dietary FODMAPs influence symptoms in irritable bowel syndrome (IBS). Poor absorption of high-FODMAP carbohydrates in the small intestine allows fermentable substrates to reach the colon, where bacterial fermentation increases gas production, luminal water content, and intestinal distension, thereby exacerbating symptoms. In contrast, a low-FODMAP diet reduces the delivery of fermentable substrates to the colon, leading to decreased fermentation and improved gastrointestinal symptoms. Created in BioRender. Vukojevic, K. (2026) https://BioRender.com/ebb9a5v (accessed on 10 November 2025).

A variety of other diets have been proposed for IBS, though the evidence is less robust than for the low FODMAP diet. A gluten-free diet (GFD) is tried by some IBS patients, especially those without celiac disease but who suspect non-celiac gluten sensitivity. Small trials suggest that a subset of IBS-D patients may improve on a GFD, with one study reporting symptom improvement, particularly among those with the HLA-DQ2/8 genotype (associated with celiac), despite negative celiac tests [44]. It is unclear if the benefit is due to the removal of gluten or the concurrent reduction in FODMAPs (wheat contains both). Current guidelines generally reserve GFD for those with clear gluten sensitivity or as a second-line trial if FODMAP reduction fails [1]. Lactose-free diets can help a considerable fraction of IBS patients with lactase deficiency; lactose is a FODMAP disaccharide, and its malabsorption can cause gas and diarrhea. Simply avoiding high-lactose dairy (or using lactase enzyme supplements) may be sufficient for those individuals. The Specific Carbohydrate Diet (SCD) and Paleo diets have anecdotal support but lack strong research in IBS; they similarly restrict certain carbohydrates but are less systematically studied. For SIBO, one dietary approach is the elemental diet, which involves consuming an easily absorbed, pre-digested liquid formula (containing amino acids, glucose, and essential nutrients) for 2–3 weeks. The elemental formula provides nutrition while theoretically starving the bacteria in the small bowel, since simple sugars are absorbed rapidly in the upper gut and little is left for microbes. A small study reported that a 14-day elemental diet achieved ~80% normalization of the breath test in known SIBO patients [45]. While effective in some cases, the elemental diet is challenging due to palatability and cost, and it should be medically supervised. It may be considered for refractory SIBO or when antibiotics are contraindicated. Finally, general healthy-diet principles—such as maintaining regular meal patterns, limiting excess fat, caffeine, and spicy foods—are often recommended for individuals with IBS. Eating smaller, more frequent meals can help prevent gut overload. Trigger foods (certain vegetables, high-fat meals, alcohol) should be identified by each patient and moderated. Adequate water intake and regular physical activity also support bowel regularity as part of a lifestyle management plan [37,46,47]. As illustrated in Figure 2, reducing dietary FODMAP intake limits the delivery of fermentable substrates to the colon, thereby decreasing bacterial fermentation, gas production, and luminal distension, which contributes to symptom relief in patients with IBS.

### 4.2. Probiotics and Prebiotics

Given the evidence of microbiota disturbances in IBS and SIBO, probiotics—live microorganisms that, when administered in adequate amounts, confer a health benefit—have been extensively studied as a therapeutic option. Many IBS patients show interest in probiotics as a “natural” treatment to restore a healthy gut flora. A large number of randomized controlled trials have evaluated various probiotic strains and combinations in IBS, with overall results indicating a modest but significant benefit for global symptoms and abdominal pain, particularly with multi-strain formulations [29,47]. A comprehensive meta-analysis of 53 RCTs involving over 5500 IBS patients found that probiotics as a class improved the probability of IBS symptom relief compared to placebo (RR for remaining symptomatic ~0.79, meaning probiotics provided benefit) [29,47]. Specific improvements were noted in bloating and flatulence scores, and certain strains showed benefit for abdominal pain severity [29]. For example, combinations of Bifidobacterium and Lactobacillus species have frequently been reported to alleviate IBS symptoms. A multi-center trial of a *Bifidobacterium bifidum* strain demonstrated a significant improvement in IBS symptom scores compared to a placebo [48]. The mechanisms by which probiotics may help include: modulation of the gut microbiota composition (e.g., increasing beneficial bacteria, reducing gas-producing species), enhancement of the intestinal barrier function, competition with or suppression of pathogenic bacteria, and even direct effects on gut sensory and motor functions (through microbial metabolites or interaction with neural pathways) [49,50]. Probiotics can exert anti-inflammatory effects; for instance, probiotic interventions in IBS have been shown to reduce pro-inflammatory mediators (e.g., IL-6, TNF-α, CRP) and fecal inflammatory markers, alongside improving barrier function in the colon [51].

However, not all probiotics are equal, and identifying the optimal species/strains for IBS remains challenging. Trials are heterogeneous; different probiotic formulations, doses, and durations have been tested, with varying outcomes. A 2022 network meta-analysis suggested that *Bacillus coagulans* might have the highest probability of being the most effective single species for IBS, improving global symptoms and pain; however, it also confirmed that many probiotics have overlapping efficacy and that few direct comparisons exist [52]. Multi-strain products (combining, say, 8–10 strains) are commonly used in practice, aiming for a broader effect. Current evidence supports a trial of probiotics in IBS, acknowledging that individual responses may vary. The American College of Gastroenterology’s latest guideline (2021) takes a somewhat cautious view due to trial heterogeneity. Still, other experts and a recent British guideline consider probiotics a reasonable option for managing IBS symptoms [42]. Importantly, probiotics are generally safe and well-tolerated; adverse events are rare and often similar to those of a placebo (mild gastrointestinal upset in a minority).

In the context of SIBO, one might assume that adding bacteria (probiotics) could worsen an overgrowth scenario. Interestingly, several studies have explored the use of probiotics for SIBO, yielding some positive results. The rationale is that probiotic organisms might suppress or outcompete the abnormal overgrowth, enhance gut immunity, or improve motility (some probiotics produce substances that stimulate migrating motor complexes). A 2017 systematic review and meta-analysis of probiotics in SIBO [53]) found that probiotics were associated with SIBO eradication (as measured by breath test normalization) in some studies and with symptom improvement. For instance, one trial demonstrated that *Lactobacillus* probiotics administered for 4 weeks resulted in symptom relief and a decrease in hydrogen breath gas levels in SIBO patients [54]. Probiotics have also been used as adjuncts to antibiotics to prevent post-treatment SIBO recurrence. While the evidence is not as robust as in IBS, probiotics may benefit SIBO patients, especially in preventing relapse after antibiotic clearance [53]. Care must be taken in specific scenarios (such as severe immunosuppression or short bowel syndrome with stagnation), as probiotics in rare cases can translocate and cause infection. Still, in the typical SIBO patient, this is exceedingly uncommon.

Prebiotics are fermentable fibers or compounds that selectively stimulate the growth or activity of beneficial gut bacteria primarily in the colon. Examples include inulin, fructo-oligosaccharides (FOSs), galacto-oligosaccharides (GOSs), and resistant starches. In IBS, prebiotics present a double-edged sword: while they can boost populations of health-promoting microbes, such as Bifidobacteria, they also produce gas during fermentation, which may exacerbate bloating and pain if introduced too quickly or in high amounts. Clinical data on prebiotics in IBS are limited and mixed. Some small studies using GOS (which feeds *Bifidobacteria*) have shown improvements in bloating and anxiety in IBS patients on low doses (≤3.5 g/day), presumably by modulating the gut–brain axis [55]. A systematic review found insufficient evidence that prebiotics alone significantly improve IBS symptoms [30], and they are not routinely recommended as a front-line therapy. But previously mentioned partially hydrolyzed guar gum (PHGG), a soluble fiber with prebiotic properties, has garnered interest because it ferments more slowly and tends to be better tolerated. PHGG has garnered interest due to its slow fermentation profile and superior tolerability compared to other soluble fibers. In a randomized controlled trial, a 12-week administration of 6 g/day of PHGG significantly improved bloating and gas scores compared to placebo, with benefits persisting for four weeks post-treatment [56]. Prebiotics may be more useful in constipation-predominant IBS, where increasing SCFA production and water content can help bowel movements.

Synbiotics, which combine probiotics and prebiotic fibers, are another approach—the prebiotic feeds the administered probiotic or native good bugs. A few trials of synbiotics in IBS have shown symptom improvements (e.g., a combination of *Bifidobacterium* plus inulin reducing IBS symptom scores), but again, data are scarce. Considering the overall picture, a practical strategy is often to start with probiotics (to introduce beneficial strains) and later add soluble fiber or prebiotics carefully as tolerated, rather than large doses of fermentable prebiotics upfront [57,58].

### 4.3. Herbal and Nutraceutical Therapies

A variety of herbal and nutraceutical treatments have been explored for IBS and SIBO, often with the appeal of being “natural” alternatives to pharmaceuticals. Among these, peppermint oil stands out as one of the most well-supported therapies for IBS. Peppermint oil (derived from *Mentha piperita*) contains menthol and other compounds that have antispasmodic effects on intestinal smooth muscle by blocking calcium channels [59]. Multiple randomized trials and meta-analyses have confirmed that peppermint oil can significantly reduce abdominal pain and improve overall IBS symptoms compared to a placebo [46,47]. A 2008 meta-analysis by Ford et al. reported a relative risk of 0.43 for persistent IBS symptoms with peppermint oil compared to placebo, indicating that patients were more than twice as likely to experience symptom relief with peppermint oil [46]. Peppermint oil is now recommended in many guidelines as an effective and safe treatment for IBS, particularly for alleviating pain and bloating [52]. Typical dosing is 180–225 mg of peppermint oil per capsule, taken two or three times daily before meals. Side effects are minimal, mostly a transient minty sensation or heartburn in some if the coating dissolves too early. However, a recent multicenter pediatric RCT found that peppermint oil capsules (and peppermint sweets) were not superior to placebo for abdominal pain reduction or adequate relief in children with IBS or functional abdominal pain [60]. Overall, peppermint oil remains an evidence-based, non-prescription antispasmodic option for IBS, with the strongest evidence in adults.

For SIBO, herbal antimicrobials have gained attention as alternatives to antibiotics such as rifaximin. Botanical combinations (often containing herbs with antimicrobial properties such as oregano oil, garlic extract [allicin], thyme, berberine from plants like Berberis or Goldenseal, neem, and others) are used to suppress bacterial overgrowth. A notable study by Chedid et al. [61] provided one of the first comparisons: in a randomized trial of SIBO patients, a specific herbal supplement regimen (containing oregano, thyme, berberine, etc.) was found to be as effective as rifaximin in eradicating SIBO based on breath test results (approximately 46% vs. 34% eradication; difference not statistically significant). Additionally, some patients who had failed rifaximin subsequently responded to the herbal therapy [61]. This study suggests that herbal combinations can achieve SIBO clearance in a significant proportion of cases, offering a viable alternative, especially for those who prefer to avoid antibiotics or have rifaximin-resistant SIBO. Another example is berberine, an alkaloid found in plants such as *Berberis vulgaris*. Berberine has broad antimicrobial effects and some prokinetic activity. An ongoing trial (the BRIEF-SIBO study) directly compares berberine vs. rifaximin in SIBO [62]. While results are pending, berberine is already used empirically by some practitioners, usually at doses around 500 mg two to three times daily for 4–6 weeks. Caution is warranted as berberine can cause constipation or low blood pressure in some, and robust safety data beyond short courses are limited.

Other nutraceuticals with evidence for IBS include IBerogast (STW-5), a liquid herbal extract blend (including chamomile, caraway, peppermint, licorice, and others) with antispasmodic and pro-motility effects. Some trials and a meta-analysis indicated Iberogast can improve IBS symptoms, particularly pain and bloating, versus a placebo [63]. It is available over the counter in many countries. Artichoke leaf extract has also been reported in an open-label study to reduce dyspepsia and IBS symptoms, though placebo-controlled data are lacking [64].

From a symptom standpoint, certain herbs target specific issues: caraway and fennel have carminative (gas-reducing) properties; ginger may aid gastric emptying and overall motility; and turmeric has been studied for dyspepsia/IBS, with some benefit attributed to its anti-inflammatory effects. While these are not mainstream, patients often try dietary supplements like turmeric or ginger for adjuvant relief, and some small studies support their use [65,66,67,68].

Glutamine is worth mentioning as a nutraceutical for gut health, especially in post-infectious or diarrhea-predominant IBS, where increased intestinal permeability is suspected. Glutamine is a key fuel for enterocytes and can help restore tight junction integrity. A randomized controlled trial in IBS-D patients with evidence of leaky gut found that oral glutamine (5 g TID) for 8 weeks significantly improved IBS symptoms and reduced intestinal permeability compared to placebo [69]. This suggests glutamine supplementation can ameliorate IBS symptoms by enhancing the gut barrier, potentially useful in IBS patients with concurrent SIBO or inflammation that might impair barrier function. Although more research is needed, glutamine is a relatively safe amino acid supplement, and some clinicians incorporate it (doses ~10–15 g per day, divided) for IBS-D or PI-IBS cases.

Enzyme supplements (like lactase for lactose intolerance or alpha-galactosidase for gas-producing oligosaccharides in beans) can be considered on an as-needed basis to help digest specific trigger foods rather than avoiding them entirely. For example, lactase enzyme drops or tablets allow many lactose-intolerant IBS patients to consume dairy without symptoms [70,71].

Finally, it should be noted that acupuncture and related traditional Chinese medicine approaches have been explored for IBS. Results are mixed: some studies show acupuncture can improve IBS symptoms and quality of life, possibly by modulating visceral pain pathways and autonomic function, but sham-controlled trials often demonstrate large placebo effects. A recent systematic review [72] concluded that true acupuncture was not significantly better than sham acupuncture in IBS, although both produced improvement over no treatment, highlighting a strong placebo/expectancy component. Despite limited evidence, acupuncture remains a popular alternative therapy among patients seeking holistic care, and some individuals report benefit in symptom relief and stress reduction, which can indirectly help IBS.

### 4.4. Psychosocial and Mind–Body Interventions

Psychological stress and psychosocial factors play a substantial role in the exacerbation and maintenance of IBS symptoms via the brain–gut axis. Many patients note that stress worsens their GI symptoms, and there are high rates of anxiety, depression, or a history of trauma in IBS populations [73]. Consequently, brain–gut therapies are a cornerstone of IBS management, addressing the central nervous system aspects of the disorder. The brain-gut-microbiota axis operates bidirectionally: psychological stress can alter gut microbiota composition and motility patterns, while gut dysbiosis and inflammation can influence central pain processing and mood [9,10,27,28]. Impaired gastrointestinal motility, particularly dysfunction of the migrating motor complex, is a well-established risk factor for SIBO development [16,17], and stress-related autonomic dysregulation may contribute to these motility disturbances. Mind–body interventions work by modulating autonomic nervous system function and central pain processing, potentially restoring normal gut motility patterns and reducing the visceral hypersensitivity that amplifies symptoms in both IBS and SIBO [9,10].

Cognitive behavioral therapy (CBT) is a structured, skills-based psychotherapy that helps patients identify and modify maladaptive thoughts and behaviors related to their symptoms and stress. In IBS, CBT often focuses on coping strategies for pain and bowel unpredictability, stress management, and reducing catastrophic thinking about symptoms. Multiple RCTs and meta-analyses have established that CBT leads to significant improvements in IBS symptoms and quality of life compared to usual care or wait-list controls [74]. For example, a 2020 meta-analysis by Black et al. that updated prior evidence found psychological therapies, including CBT, were associated with a notable reduction in IBS symptom severity (odds of symptom persisting were ~0.6 with therapy vs. without) [74]. CBT can be delivered in various formats, including individual, group, telephone, or internet-based sessions, and even minimal-contact CBT has shown efficacy, which is promising for accessibility [74]. National IBS treatment guidelines often recommend adding CBT or other psychological therapy for patients with moderate-to-severe IBS or those who have not responded to dietary/pharmacologic measures [1].

Gut-directed hypnotherapy uses hypnotic suggestion and relaxation techniques to target gut function and sensation specifically [75]. During sessions, a hypnotherapist guides the patient into a relaxed, focused state and provides positive suggestions to normalize GI motility, reduce pain sensitivity, and improve gastrointestinal well-being. Pioneered by Dr. Peter Whorwell and colleagues, gut-directed hypnotherapy (often called gut-focused or IBS hypnotherapy) has been validated in multiple trials. A landmark study showed that approximately 70–80% of IBS patients responded to a course of hypnotherapy, with sustained symptom relief, and these improvements often lasted for years after treatment [76,77]. More recent trials, including a large multicenter trial in 2019 [76], confirmed that hypnotherapy (both individual and group formats) leads to significant reductions in IBS symptom severity and can be as effective as CBT. Hypnotherapy is now considered an effective therapy for IBS by organizations like the American Gastroenterological Association. Access to trained hypnotherapists can be a limiting factor, but the rise of audio-recorded sessions and home-based protocols is expanding their reach [77].

Beyond formal psychotherapy, general stress reduction can meaningfully impact IBS. Techniques such as mindfulness meditation, progressive muscle relaxation, and deep breathing exercises activate the parasympathetic nervous system, thereby dampening the stress response, which in turn can help calm GI motility and sensitivity. Mindfulness-based stress reduction (MBSR) programs have shown moderate benefits in improving IBS symptoms and reducing psychological distress in some trials [78]. Even yoga, which combines physical postures with breathing and mindfulness, has been studied: a randomized trial found that a yoga program was comparable to a low FODMAP diet in reducing IBS symptom scores, highlighting the gut–brain connection [79]. Regular exercise is generally recommended for IBS—aerobic exercise has been shown to decrease symptom severity, possibly by improving gastrointestinal transit and reducing stress [80]. Patients with IBS are encouraged to engage in consistent moderate physical activity (20–30 min, 3–5 times a week), which can help alleviate constipation, improve mood, and enhance overall gut function [81].

In more severe cases or when a significant mood disorder coexists, involvement of mental health professionals and use of psychotropic medications used primarily for central neuromodulatory effects may be appropriate. Antidepressants, particularly low-dose tricyclic antidepressants or SNRIs, can modulate pain signaling in IBS and are often considered when pain is prominent [81,82,83]. Clinicians may prescribe these; however, they often emphasize the interplay of psychological and physical in IBS treatment [42,81,82]. Also, there are more emerging studies on antidepressants’ antimicrobial activity. A large population-based shotgun metagenomics study, use of several non-antibiotic drug classes—including antidepressants—and polypharmacy, were associated with changes in gut microbial species, supporting the concept that chronic medication exposure can modulate microbiota [83]. This was in line with the previous in vitro study that has shown that multiple antidepressants can exhibit direct antimicrobial activity against members of the normal gut microbiota [84], raising the possibility that, in susceptible individuals, psychotropic treatment could contribute to dysbiosis-like changes. Therefore, when antidepressants are considered as part of IBS management, it may be clinically relevant to recognize potential microbiome effects—particularly in patients with recurrent dysbiosis/SIBO or frequent antibiotic exposure—while balancing these considerations against their demonstrated benefits for pain modulation and comorbid anxiety/depression.

In summary, addressing the mental health and stress component is a critical pillar of IBS management. These interventions modulate the brain-gut-microbiota axis [9,10,27], addressing not only symptom perception but also underlying dysfunction in gut motility and immune signaling that may predispose to or perpetuate both IBS and SIBO [16,17]. While these interventions do not eradicate SIBO if present, they can improve a patient’s tolerance of visceral stimuli and break the cycle wherein anxiety or stress exacerbates GI symptoms, which in turn creates more anxiety. The ideal IBS treatment plan often combines dietary modifications (to reduce peripheral triggers, such as gas), microbiota modulation (if dysbiosis or SIBO is a factor), and psychosocial therapies (to recalibrate the brain–gut axis). This comprehensive approach yields the best chance of sustained symptom control.

### 4.5. Fecal Microbiota Transplantation (FMT)

One of the most direct ways to alter the gut microbiome is fecal microbiota transplantation, the process of infusing a processed stool sample from a healthy donor into the patient’s gastrointestinal tract to “reset” the microbiota. FMT is an established, highly effective treatment for *Clostridioides difficile* infection, and its success there has prompted exploration in other conditions involving dysbiosis, including IBS. The rationale in IBS is that introducing a diverse, balanced microbial community from a healthy individual might correct the dysbiotic ecosystem of the IBS patient’s gut, thereby alleviating symptoms.

Clinical trials of FMT in IBS have yielded mixed results, highlighting the importance of factors such as donor selection, route of administration, and patient subtypes. Two early placebo-controlled trials reached opposite conclusions: one (in capsules) showed no significant benefit of FMT over placebo in moderate IBS [31], while another (via colonoscopy enema) found marked improvement in IBS symptoms for FMT recipients vs. placebo, especially when using a “super-donor” with a very healthy microbiome [30]. El-Salhy et al. reported response rates of 77% with a 30 g FMT and 89% with a 60 g FMT, compared to 24% with a placebo (own feces), along with significant increases in microbial diversity in the treated groups [30]. Consistent with this rationale, microbiome analyses in IBS trials show post-FMT engraftment patterns with enrichment of taxa commonly regarded as beneficial, including increased *Lactobacillus* spp. in responders and enrichment of *Bifidobacterium* (e.g., B. longum) after FMT [30,85].

These impressive results suggest FMT can be highly effective in some IBS patients, who may be IBS-D, C or M types. However, other studies have been less successful or have yielded only modestly positive results. A recent meta-analysis of FMT in IBS (aggregating five RCTs) found that FMT overall led to improvement in IBS severity and quality of life, but with considerable heterogeneity between trials [85]. Notably, the choice of donor seems crucial—“super-donors” with specific beneficial microbiota profiles may be required for optimal results (and the El-Salhy trial used stool from a single donor with a high relative abundance of certain favorable bacteria). The route may also matter: lower delivery (colonic) might ensure that more transplanted bacteria colonize the large bowel, whereas upper delivery or capsules must survive passage and may be better suited for the small intestine, although data are sparse.

As of now, FMT for IBS remains investigational and is not an approved or routine therapy. Safety in IBS appears acceptable in the short term (transient mild GI side effects are common; serious adverse events are rare in trials, but there are isolated reports of weight gain or new conditions possibly triggered by FMT, underscoring the need for caution and screening). Given inconsistent data, guidelines do not currently recommend FMT for IBS outside of research settings [86]. But research is ongoing, and FMT could become a targeted therapy in the future if we learn how to select ideal donor microbiomes and identify which IBS patients are likely to respond (e.g., perhaps those with specific baseline dysbiosis markers). Additionally, refined approaches, such as defined microbial consortia (utilizing selected strains instead of whole stool), are being explored to achieve the benefits of FMT with greater control and safety [85,87].

While FMT has historically been viewed as a peripheral consideration for SIBO—largely because it delivers microbes to the colon rather than the small intestine—recent data suggest its impact is more systemic than previously assumed. Notably, the presence of baseline SIBO has been shown to actually enhance the clinical efficacy of FMT given as infusion via nasogastric tube in patients with chronic constipation, resulting in superior colonic motility and symptom relief compared to those without overgrowth [88]. This shift from anecdotal observation to clinical evidence is further supported by randomized, placebo-controlled data demonstrating that FMT, also given in the form of oral capsules, can effectively eradicate SIBO, normalize breath tests, and reestablish microbial diversity [89]. Consequently, while SIBO management has traditionally focused on reducing bacterial load, these findings suggest that “resetting” the small intestinal environment via FMT may offer a novel therapeutic pathway, though further evidence is required before it can be integrated into routine practice.

## 5. Conclusions

IBS and SIBO are conditions that epitomize the complex interplay between the gut’s physiology, its microbial inhabitants, and the nervous system. The pathophysiology of IBS involves multiple factors, including disordered motility, visceral hypersensitivity, immune activation, and dysbiosis, that manifest as chronic GI symptoms. While the precise causal relationship between SIBO and IBS remains unclear and likely varies among patients, these conditions frequently co-occur and share overlapping pathophysiological mechanisms. Whether SIBO acts as a trigger, consequence, or parallel phenomenon in IBS, the intestinal microbiota clearly plays a significant role in generating and maintaining symptoms in both conditions. Recognizing these mechanisms broadens the therapeutic paradigm beyond conventional pharmacotherapy to a more holistic management strategy [17].

Non-pharmacological interventions form a cornerstone of this comprehensive approach. Dietary modification, especially the low FODMAP diet, has demonstrated substantial efficacy in reducing IBS symptoms by limiting fermentable substrates that feed gas-producing gut microbes. Tailoring fiber intake, by boosting soluble fiber and avoiding insoluble fiber that can be irritating, helps regulate bowel habits and improve tolerability [90,91]. Concurrently, probiotics aim to recalibrate the microbiota and have shown promise in mitigating IBS symptom severity, as well as possibly preventing SIBO recurrence [91,92,93,94]. Herbal therapies, such as peppermint oil and botanical antimicrobial compounds, offer additional tools to relieve symptoms and combat bacterial overgrowth, often with favorable safety profiles [46,58,59,60,64]. Critically, mind–body interventions address the central drivers of IBS, leveraging techniques like CBT and hypnotherapy to dampen the brain–gut dysregulation and break the stress–symptom cycle. These psychological therapies have outcomes comparable to medical treatments for many patients and underscore the importance of treating the “brain” side of the disorder of gut–brain interaction [73,74,75,76,77,95].

The integration of gut-focused and brain-focused therapies is compelling. For example, combining a dietary intervention to reduce peripheral triggers, a probiotic to normalize the microbiome, and CBT to build stress resilience can act synergistically to provide relief greater than any single modality alone. This multimodal strategy is often necessary given the multifaceted nature of IBS. In the case of SIBO, antibiotic therapy (rifaximin or others) is frequently needed to reduce bacterial load initially, but non-pharmacological measures such as dietary adjustments (to prevent regrowth), prokinetics or physical activity (to enhance motility), and possibly probiotics (to maintain a healthier flora) are critical for long-term success and relapse prevention [96,97]. Indeed, one of the challenges in SIBO management is the high recurrence rate after antibiotics; a holistic plan, including diet and perhaps herbs or probiotics, is recommended to extend remission.

Despite clinical overlap and shared therapeutic targets, evidence for a direct causal link between SIBO and IBS remains limited. Whether SIBO triggers, results from, or coexists with IBS is unclear. Our integrated approach reflects that interventions targeting shared mechanisms can benefit patients regardless of which condition predominates. As we move towards more personalized medicine, understanding an individual patient’s predominant symptom triggers and pathophysiological profile (e.g., whether dysbiosis is prominent, whether stress is a major exacerbating factor, or whether diet is a key trigger) allows for tailoring non-pharmacological interventions to those most likely to be beneficial. For instance, a patient with IBS-D and a positive breath test might receive a low-FODMAP diet, a rifaximin course followed by probiotics, and gut-directed hypnotherapy targeting the microbiota, diet, and brain–gut axis concurrently. Another patient with IBS-C might focus on soluble fiber supplementation, peppermint oil for pain, and a yoga-based stress reduction program. Both may achieve significant improvement through different pathways [98,99].

Looking ahead, as research continues to unravel the complexity of the gut microbiome, we anticipate further refining these therapies. Diets may become more individualized based on one’s microbiota or metabolite profile; next-generation probiotics (or postbiotics) could more specifically correct IBS-related dysbiosis; and FMT or microbial consortia might eventually find a clear role in a subset of patients once optimal protocols are defined. Likewise, digital therapeutics delivering CBT or hypnotherapy via apps could expand access to effective brain–gut treatments. The common thread is that a biopsychosocial approach—addressing diet, microbes, and the mind—is essential for effective management of IBS, with or without SIBO [100]. By combining non-pharmacological interventions with judicious use of medications when needed, clinicians can achieve a more complete and lasting relief for patients. The comprehensive approach outlined in this review emphasizes empowering patients with self-management strategies (dietary changes, stress management techniques) alongside medical therapies, thereby improving not just symptoms but overall quality of life. In conclusion, whether approached as distinct conditions or as overlapping manifestations of gut–brain-microbiota dysfunction, IBS and SIBO necessitate an integrative treatment model, with non-pharmacological therapies at its core, providing safe and often empowering means for patients to manage and alleviate their condition.

## Figures and Tables

**Table 1:** Comparison of Pathophysiological Mechanisms and Microbial Changes in IBS and SIBO.

Aspect	IBS	SIBO	Intersection	Refs.
Core Pathophysiology	Multifactorial: brain–gut axis dysregulation, abnormal GI motility, visceral hypersensitivity, immune activation, dysbiosis	Abnormally high bacterial load in the small intestine, often colonic-type bacteria; impaired motility and host defenses	Unclear if SIBO triggers or results from IBS; both involve dysmotility and dysbiosis	[3,9,10]
Motility Abnormalities	Accelerated (IBS-D) or delayed (IBS-C) transit; post-infectious changes	Impaired migrating motor complex; risk factors: diabetes, scleroderma, PPI use, surgeries	Dysmotility in IBS may predispose to SIBO	[3,17]
Immune Activation	Low-grade mucosal inflammation (↑ mast cells, T lymphocytes); post-infectious IBS	Mucosal inflammation, brush border injury, malabsorption	Bacterial metabolites/endotoxins in SIBO can trigger IBS-like inflammation	[3,11,12,17]
Microbiota Alterations	Reduced diversity, ↓ *Bifidobacteria*/*Lactobacilli*, ↑ *Proteobacteria*/*Firmicutes*, altered SCFA profiles	Overgrowth of colonic-type aerobes/facultative anaerobes (*E. coli*, *Klebsiella*, etc.); ↑ methanogens in methane SIBO	Methane-producers linked to constipation, H_2_S-producers to diarrhea	[3,17,22,25]
Gas Production & Symptoms	Bloating, pain, altered stool form; linked to gas (H_2_, CH_4_, H_2_S) from fermentation	Excess fermentation: H_2_ (diarrhea), CH_4_ (constipation), H_2_S (diarrhea); gas type correlates with symptoms	Methane-positive breath tests correlate with IBS-C; H_2_S with IBS-D	[3,4,5,17,18]
Intestinal Permeability	Increased (“leaky gut”) in some IBS; may drive neural sensitization	Brush-border injury, mucosal inflammation, and malabsorption; may increase permeability	Both may feature barrier dysfunction	[26]
Brain–Gut–Microbiota Axis	Microbiota modulate immune, barrier, neural, and psychological function; stress can alter microbiota	SIBO may affect the CNS via immune/neural pathways	Dysbiosis may mediate stress and visceral hypersensitivity	[28,29]
Therapeutic Implications	Diet, probiotics, antibiotics (rifaximin for IBS-D), FMT (mixed results)	Antibiotics (rifaximin), prokinetics, dietary modification	Microbiota modulation is a shared therapeutic target	[30,31]

## Data Availability

No new data were created or analyzed in this study. Data sharing is not applicable to this article.
